# New insights into the construction of wild-type Saba pig-derived *Escherichia coli irp2* gene deletion strains

**DOI:** 10.1007/s13205-021-02951-0

**Published:** 2021-08-13

**Authors:** Bo Zhang, Hongdan Wang, Weiwei Zhao, Chunlan Shan, Chaoying Liu, Libo Gao, Ru Zhao, Pingxing Ao, Peng Xiao, Longbao Lv, Hong Gao

**Affiliations:** 1 College of Animal Science and Technology, Yunnan Agricultural University, Kunming, 650201 Yunnan China; 2 College of Veterinary Medicine, Yunnan Agricultural University, Kunming, 650201 Yunnan China; 3 College of Food Science and Technology, Yunnan Agricultural University, Kunming, 650201 Yunnan China; 4 Institute of Zoology, Chinese Academy of Sciences, Kunming, 650223 Yunnan China

**Keywords:** CRISPR/Cas9, *Escherichia coli*, Gene editing, *irp2* gene, Iron absorb

## Abstract

To construct wild-type *E. coli irp2* gene deletion strains, CRISPR/Cas9 gene editing technology was used, and the difficulty and key points of gene editing of wild-type strains were analyzed. Based on the resistance of the CRISPR/Cas9 system expression vector, 4 strains of 41 *E. coli* strains isolated from Saba pigs were selected as the target strains for the deletion of the *irp2* gene, which were sensitive to both ampicillin and kanamycin. Then, CRISPR/Cas9 technology was combined with homologous recombination technology to construct recombinant vectors containing Cas9, sgRNA and donor sequences to knock out the *irp2* gene. Finally, the absence of the *irp2* gene in *E. coli* was further verified by iron uptake assays, iron carrier production assays and growth curve measurements. The results showed that three of the selected strains showed single base mutations and deletions (Δ*irp2-1*, Δ*irp2-2* and Δ*irp2-3*). The deletion of the *irp2* gene reduced the ability of *E. coli* to take up iron ions and produce iron carriers, but not affect the growth characteristics of *E. coli*. It is shown that the CRISPR/Cas9 knock-out system constructed in this study can successfully knock out the *irp2* gene of the wild-type *E. coli*. Our results providing new insights into genome editing in wild-type strains, which enable further functional studies of the *irp2* gene in wild-type *E. coli*.

## Introduction

Saba pig is an excellent local breeding pig in Yunnan province in China, with wide distribution and large quantity, and is listed as a national livestock and poultry genetic resource protection breed (Lian et al. 2006). For a long time, due to inadequate feeding management and disease prevention and control, diarrhea and death caused by pathogenic *E. coli* infection in Saba pigs has become an important factor seriously threatening the development of characteristic pig industry in Yunnan province. The pathogenicity and virulence of *E. coli* are related to the high pathogenicity island (HPI), and the pathogenicity and virulence of *E. coli* are enhanced due to its existence (Rakin et al. 2013). Studies have shown that HPI has the characteristics of horizontal transfer, which has been found in different *Enterobacteriaceae* (Tu et al. 2017; Messerer et al. 2017; Benedek and Schubert 2007). HPI has a 30.5 kb functional core regions and carries *irp1*-*irp9* genes related to iron element syntheses, regulation and uptake. *Irp2* as a marker gene of HPI is an iron-regulated gene, which is only expressed in pathogenic bacteria and plays an important role in the syntheses of iron carriers and synthesis yersiniabactin (Ybt) (Tu et al. 2016; Magistro et al. 2017). *Irp2* expresses the high molecular weight protein 2 (HMWP2), which is mainly involved in siderophore and induces the expression of ironophore. *E. coli* have the ability to acquire iron from host species, and this ability is a major influence factor on their pathogenesis. Some reports have shown that deletion of *irp2* in *E. coli* abrogated Ybt production, reduced its ability to adhere to cells, and the LD50 value showed a reduction in its virulence (Smati et al. 2017; Tu et al. 2016). Therefore, it has potential values and significance to explore the relationship between iron uptake ability and *irp2* gene in *E. coli*.

The CRISPR/Cas9 system composed of clustered regularly interspaced short palindromic repeats (CRISPR) and its affiliated Cas9 protein that exists on some bacteria and most archaic (Wiedenheft 2013). When foreign genes invade bacteria and archaic, they identify the invading DNA and insert it between the leader and repeat sequences of the CRISPR sequence, while the protospacer adjacent motif (PAM) to help *Cas9* nuclease finds the cleavage site of the invading DNA, Cas9 protein then cleaves the foreign DNA to degrade it to protect the bacteria from harm (Hsieh-Feng and Yang 2020). Subsequently, the system was artificially modified with CRISPR/Cas9 gene editing technology (Demirci et al. 2018), which is simple, efficient and accurate compared to zinc finger nuclease (ZFN), transcriptional activator-like effector nuclease (TALENS) and homologous recombination technology (Urnov et al. 2010). It can be widely used in animals (Yang et al. 2018), plants (Zhang et al. 2017; Feng et al. 2013) and microorganisms (Cho et al. 2017) gene research. In CRISPR/Cas9 gene editing system, Cas9 protein is reflected on the double-strand break in DNA. If the broken gene is not repaired, the cell will die, which is one of the important factors that limit the effective function of the system. Most prokaryotic lack double-stranded DNA repaired system. When using CRISPR/Cas9 system to edit *E. coli* genes, the donor homologous to the target gene needs to be added to the vector as a template for repairing DNA double-strand breaks (DSB) to make sure that broke genes can be repaired. In addition, when homologous repair donor sequences exist, the double-stranded DNA cleaved by Cas9 protein can carry out spontaneous homologous recombination, and the repair efficiency is greatly improved (Altenbuchner 2016). Therefore, this work will adopt the strategy of combining CRISPR sgRNA and donor to knock out the *irp2* gene of wild-type *E. coli*.

Although, the CRISPR/Cas9 technology has been widely and maturely applied in *E. coli*, there is still a paucity of reports on genome editing in wild-type *E. coli*, which would pose a problem for the study of wild-type *E. coli* gene functions. Therefore, this work intends to construct wild-type *E. coli* targeting the *irp2* gene knockout through CRISPR/Cas9 gene editing technology, and to explore the correlation between *irp2* gene and iron uptake capacity of wild-type *E. coli*. Finally, the key points of using CRISPR/Cas9 technology to edit wild-type *E. coli* genes are highlighted, with a view to providing some reference for using this technology to edit the genomes of the remaining wild-type pathogens.

## Materials and methods

### Materials

pEsgRNA-eGFP and pCas plasmids are relaxation plasmids, which are used in genome engineering ways. The pEsgRNA-eGFP plasmid (Biomics Biotech Company, Kunming, China) carries the ampicillin resistance gene (Ampicillin, 60 μg/ml). The plasmid pCas (Biomics Biotech Company, Kunming, China) carries kanamycin resistance gene (Kanamycin, 50 μg/ml) and can encode *Cas9* nuclease.

41 wild-type *E. coli* strains were isolated and identified from a Saba swine farm in Chuxiong autonomous prefecture of Yunnan province in China. *E. coli* DH5α strain and *CVCC*1565 strain (*irp2*^+^ bacteria) were preserved in our laboratory. Bacterial genomic DNA extraction kit, agarose gel DNA recovery kit and plasmid mini-extraction kit were purchased from Tiangen Biochemical Technology Co. Ltd. (Kunming, China). Other reagents were all domestic analytical pure products.

### Screening of *irp2* gene knock-out target strains

41 *E. coli* strains isolated from Saba pigs were screened by LB medium containing ampicillin (60 μg/ml) and kanamycin (50 μg/ml) and the DNA of the selected strains was extracted by DNA extraction kit. The *irp2* gene in wild-type *E. coli* was amplified by PCR using primers *irp2*-F: 5′-TTCCTTCAGTCGCCTGTTA-3′, *irp2*-R: 5′-CAAGCCCGACATACTCAATCT-3′ (GenBank No. BX-936398.1). Then, the PCR amplified products were identified by 1% agarose gel.

### Construction of vectors

#### Construction of pEsgRNA-*irp2* (T1, T2, and T3) plasmids

According to the design principle of sgRNA, the target site, upstream and downstream sequences of sgRNA were determined according to the results of *irp2* gene sequence using online website (http://spot.colorado.edu/~slin/cas9.html). Then, 3 target locations were identified, named sgRNA-*irp2*T1, sgRNA-*irp2*T2, and sgRNA-*irp2*T3, respectively (Table 1). The designed 3 pairs of sgRNA sequences were synthesized by the company and then connected.

**Table 1:** The sgRNA sequences of *irp2*-(T1, T2, and T3)

Name	Sequence 5′–3′ (forward/reverse)	Restriction sites
sgRNA-*irp2*T1	GATCGTCTCAGGATTCGCTGTTACCGTTTTAGAG	BamHI
	CTAGCTCTAAAACGGTAACAGCGAATCCTGAGAC	XbaI
sgRNA-*irp2*T2	GATCGATTACCAACAATTACGCGAGGTTTTAGAG	BamHI
	CTAGCTCTAAAACCTCGCGTAATTGTTGGTAATC	XbaI
sgRNA-*irp2*T3	GATCGCCTTACCCTTCGCGAGCTGTGTTTTAGAG	BamHI
	CTAGCTCTAAAACACAGCTCGCGAAGGGTAAGGC	XbaI

The underlined bases are protective bases of the restriction enzyme digestion sites

After the pEsgRNA plasmid was extracted by kit, the pEsgRNA plasmid was digested with BamHI and XbaI restriction endonucleases to form the cohesive ends GATCC and CTAGA complementary to sgRNA. Then, 3 annealed double-stranded sgRNA (*irp2*T1, *irp2*T2, *irp2*T3) were linked with plasmid pEsgRNA which was digested by XbaI and BamHI restriction enzyme. Subsequently, the linked products were transformed into *E. coli* DH5α. After culture, 3 positive clones were selected from the plates transformed by pEsgRNA-sgRNA (*irp2*T1, *irp2*T2, and *irp2*T3) for sequencing verification. Finally, the correct vectors were named pEsgRNA-*irp2*T1, pEsgRNA-*irp2*T2, and pEsgRNA-*irp2*T3.

#### Construction of pEsgRNA-*irp2* (T1, T2, and T3)-donor plasmids

To repair the *irp2* gene effectively cut by *Cas9* nuclease, the constructed pEsgRNA-*irp2* (T1, T2, and T3) vectors need to inserted into the donor sequence which is homologous to the *irp2* gene. Using *irp2* gene as the template, the right and left donor primers were designed, and the *irp2* gene was amplified by overlapping PCR. The primers information was shown in (Table 2).

**Table 2:** The primers for amplifying donor sequences

Primers	Sequence 5′–3′ (forward/reverse)	Product size(bp)	GenBank acc. no.
L1	ACGC^a^**GTCGAC**^b^GATCTGCTGCTGGCTGATCTGA(*SalI*)	471	CP058574.1
L2	*CGTACTTTCGGTCATGTTCG*^c^GATGGCACGTCTTACCGAAAAGC		
R1	*TTTCGGTAAGACGTGCCATC*^c^CGAACATGACCGAAAGTACGC	400	CP058574.1
R2	ACAT^a^**GCATGC**^b^CGGTGGCGATTGTCCG(*SphI*)		

^a^The base marked with an underline is the protective base of the corresponding restriction site

^b^The bases in bold form were restriction sites (SalI and SphI)

^c^The bases in italic format are the overlap sequences used to connect donor sequences by overlapping PCR

The genomic DNA of the target strains were used as the template for the amplification of left and right donors. L-donor was amplified by primers L1 and L2, R-donor was amplified by primers R1 and R2. After acquiring L-donor and R-donor, connect them. The ligated donor and pEsgRNA-*irp2* (T1, T2, and T3) plasmids were treated with *SalI* and *SphI* endonuclease to produce the same cohesive ends. To connected donors with the pEsgRNA-*irp2* (T1, T2, and T3) plasmids by T4-DNA ligase. All ligation products were transformed into *E. coli DH5α*, cultured overnight at 37 °C, then the positive clones were selected the next day. Subsequently, the donor sequence in the vector was amplified by PCR and verified by sequencing. Finally, the verified vectors were named pEsgRNA-*irp2*T1-donor, pEsgRNA-*irp2*T2-donor, and pEsgRNA-*irp2*T3-donor.

### *Irp2* gene knocking out by CRISPR/Cas9 gene editing

The pCas vector was transformed into target strains, and positive clones were screened by LB plate containing kanamycin. Subsequently, the constructed vectors pEsgRNA-*irp2*T1-donor, pEsgRNA-*irp2*T2-donor, and pEsgRNA-*irp2*T3-donor were transformed into the selected *E. coli* strains containing kanamycin resistance gene. There were four groups of each time-point (at 12, 24, 36, and 48 h). The first group included pCas and pEsgRNA-*irp2*T1-donor, the second group included pCas and pEsgRNA-*irp2*T2-donor, the third group included pCas and pEsgRNA-*irp2*T3-donor, the last group included pCas, pEsgRNA-*irp2*T1-donor, pEsgRNA-*irp2*T2-donor, and pEsgRNA-*irp2*T3-donor. The positive clones were screened by LB plate containing ampicillin and kanamycin. Four positive clones of each group were chosen for PCR amplification and sequencing.

### Determination of growth curves

After the target strains of *E. coli* were cultured overnight, then 200 μl culture solution was inoculated into 250 ml LB medium. The OD600 of culture was measured by ultraviolet spectrophotometer for periods of time ranging from 0 to 23 h. The growth curve of *E. coli* was plotted with time as abscissa and OD600 as ordinate.

### Detection of iron carriers

The *E. coli* strains were inoculated into LBD liquid medium (2-2′-bipyridyl 0.4 mmol/l) for night culture, take 2 ml of the bacterial solution and centrifuged at 8000 r/min, then suck 1.5 ml of supernatant and add to the same volume of chrome azurol sulphonate (CAS) detection solution. When iron ions were dissociated from the CAS complex after 1 h, the color of the CAS blue detection solution became orange-yellow. According to the ratio of the 630 nm light absorption of the CAS blue detection solution before and after the iron carrier was added, the concentration of test *E. coli* strains to produce iron carriers was determined (Schwyn and Neilands 1987). The ratio of A/Ar (Ar is the control value) as a quantitative indicator. The smaller the A/Ar ratio is, the larger the 1 − A/Ar ratio is, and the higher the yield of iron carriers is.

### Determination of iron absorption capacity of *E. coli* strains

*E. coli* strains were infected with porcine intestinal epithelial cells (IPEC-J2 cells), and a control group was established at the same time. The cells and supernatant were collected at 3, 6, 9, 12, and 24 h. After centrifugation at 3000 rpm for 5 min, 200 μl supernatant was drawn after precipitation was removed, and the concentration of iron ion in cells was determined according to the instructions of iron determination kit. The significance of data difference was analyzed using GraphPad prism 6.0 and SPSS 24.0 software.

## Results

### Screening of target strains and identification of *irp2* gene

41 strains of wild-type *E. coli* were inoculated in LB medium containing ampicillin (Ampicillin concentration was 60 μg/ml), and 10 strains were sensitive to ampicillin. 10 strains of ampicillin sensitive *E. coli* were inoculated in LB medium (kanamycin concentration was 50 μg/ml), four strains of them were sensitive to ampicillin and kanamycin. Finally, the *irp2* gene was found in four strains of *E. coli* screened by PCR amplification, which could be used as *irp2* gene knock-out target strains.

### Construction of plasmids

#### Construction of pEsgRNA-*irp2 *(T1, T2, and T3) plasmids

The pEsgRNA plasmid was linearized by double digestion with XbaI and BamHI enzymes. Then 3 sgRNAs were connected with the enzymatically digested pEsgRNA plasmid, whereafter the ligation products were transferred into *E. coli* DH5α, coated on a plate containing ampicillin and cultured overnight at 37 °C. The next day, 3 positive colonies were randomly selected from the plate to extract plasmids and sequenced, the sequence results were consistent with the original sgRNA sequence. The results showed that pEsgRNA-*irp2* (T1, T2, T3) plasmids were constructed.

#### Construction of pEsgRNA-*irp2 *(T1, T2, and T3)-donor plasmids

**Fig. 1:**
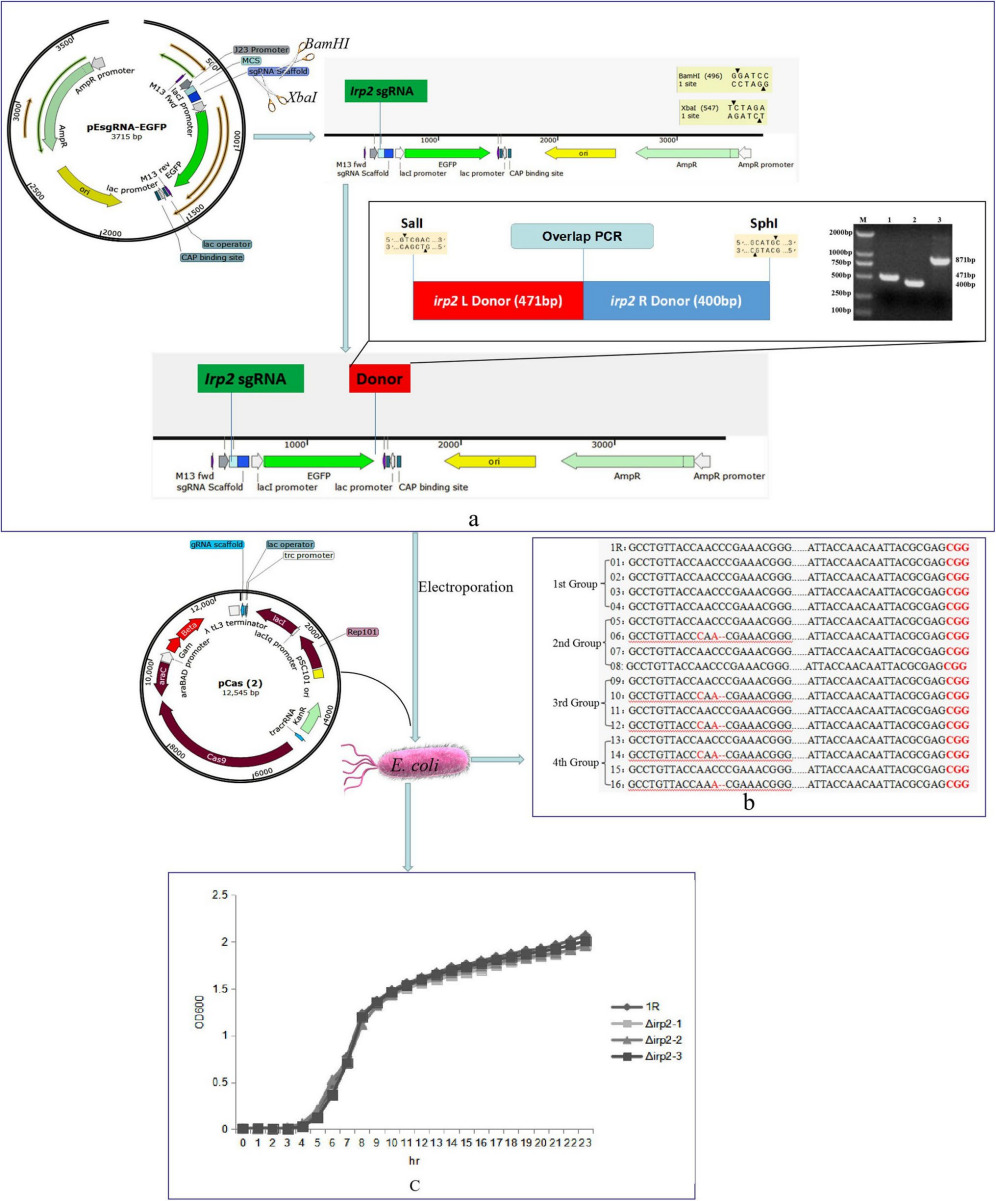
Construction of *E. coli irp2* gene deletion strains and determination of growth curves. **a** Construction of pEsgRNA-irp2 (T1, T2, and T3)-donor plasmids. **b** The sequences comparison between wild-type strain 1R and gene deletion strains. CGG (the PAM sequence). A and C (the red alphabets are mutant bases). Dashed lines (the C base deletion). **c** The growth curves of wild-type *E.coli* strain 1R and three *Δirp2* strains

The overlapping PCR method was adopted to amplify left and right donors with primers L1, L2, R1, and R2. Then the left and right donors were linked. The ligated left and right donors were digesting and inserted into the vector pEsgRNA-*irp2* (T1, T2, and T3) and transferred into *E. coli DH5α*. The positive clones were randomly selected from the resistant plate. PCR method was used to verify whether the donor was linked with pEsgRNA-*irp2* (T1, T2, and T3) (Fig. 1a).

### Construction of *irp2* gene knock-out *E. coli* strains

The two *irp2* gene editing vectors were co-electroporated into target strains. After electroporation, the positive clones were chosen by overnight culture on LB plate containing ampicillin and kanamycin. The positive clones were identified by PCR amplified and sequenced. The results indicated that the first group sequences remained. In the second group, point mutations of A → C and C → A as well as the deletion of C base were found at 1073, 1075, and 1076 of the *irp2* gene sequences in pCas and pEsgRNA-*irp2*T2-donor plasmids transferred strains. In the third group of *E. coli* transferred by pCas and pEsgRNA-*irp2*T3-donor plasmids, point mutations of A → C, C → A, and C base deletion were observed at 1073, 1075, and 1076 of the *irp2* gene sequence, respectively. C → A mutation and C base deletion occurred at 1075 and 1076 sites of *irp2* gene sequence in the last group of *E. coli* transferred by pCas, pEsgRNA-*irp2*T1-donor, pEsgRNA-*irp2*T2-donor, and pEsgRNA-*irp2*T3-donor plasmids, respectively (Fig. 1b). Finally, four strains were named as *1R* (wild-type strain), Δ*irp2-1*, Δ*irp2-2*, and Δ*irp2-3*.

### Determination of growth curves

Four strains of *E. coli* were inoculated into the liquid medium for oscillatory culture, and the optical density values of bacteria liquid were recorded in different time points. The growth curves of *E. coli* were drawn with the culture time as abscissa and optical density value of the bacterial liquid as the ordinate. The results showed that the growth trend of the strains before and after *irp2* gene knockout was the same, especially in the delayed and stable periods (Fig. 1c).

### The results of iron carrier medium detection

Four strains of *E. coli* and *CVCC*1565 strain were inoculated into the LBD iron-deficient medium. After overnight incubation, the supernatant was centrifuged and mixed with CAS blue detection liquid. After 1 h of rest, the light absorption value of the bacterial liquid was determined at 630 nm wavelength. The results showed that the 1 − A/Ar value (Ar value was 0.291) of strain *CVCC*1565 was the highest, and the ability to produce iron carriers were the highest. 1 − A/Ar values of the other 3 strains were generally decreased (Table 3). It is suggested that the deletion of *irp2* gene leads to a decrease in the ability of *E.coli* to produce iron carriers.

**Table 3:** The quantitative detection of the iron load produced by *E. coli*

Strains	A	1 − A/Ar
*CVCC*1565	0.185	0.364
*1R*	0.191	0.344
Δirp2-1	0.255	0.124
Δirp2-2	0.228	0.216
Δirp2-3	0.258	0.113

The smaller the A/Ar ratio is, the larger the 1 − A/Ar ratio is, and the higher the yield of iron carrier is. The amount of iron carriers produced by *E. coli CVCC*1565 was the largest, and the amount of iron carriers produced by Δirp2 was lower than that of wild-type *E. coli 1R*

### Effect of gene knock out on the uptake of iron by *E. coli*

**Fig. 2:**
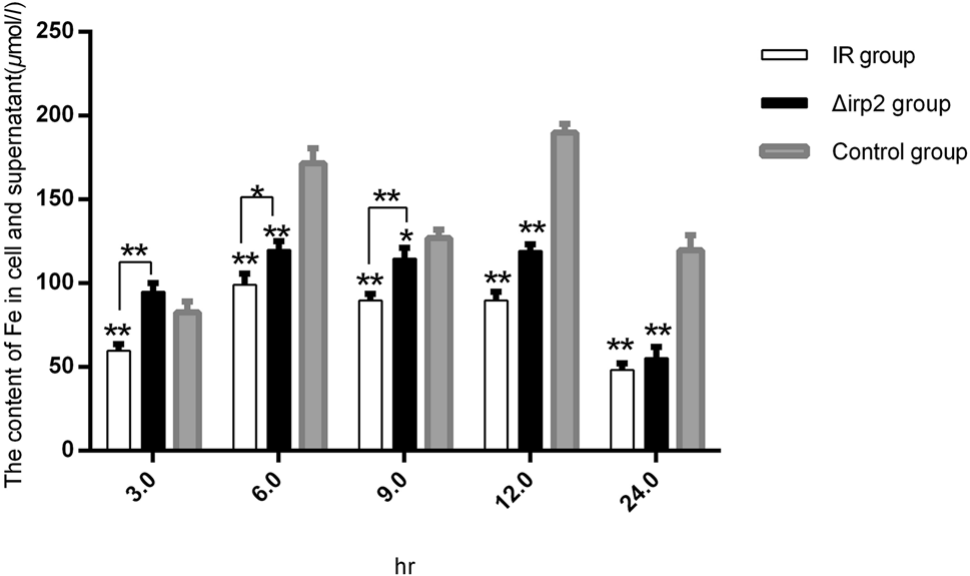
The changes of iron ion in IPEC-J2 cell and supernatant. *P* values were calculated using the Student’s *t* test. *P* < 0.05(*) or *P* < 0.01(**) was considered statistically significant

The changes of iron in IPEC-J2 cells and supernatants were measured at 5 different time points using the iron ion detection kit after 4 strains of *E. coli* was infected with IPEC-J2. The results showed that the content of iron ion in the experimental group and the control group increased at first and then decreased. The content of iron ion in group *1R* was less than that in group Δ*irp2*. The content of iron in *E. coli* infection group was less than that in control group except at the 3rd hour. The content of iron in *1R* group was significantly less than that in the control group at 5-time points (*P* < 0.01), and the content of iron *Δirp2* group was lower than that in control group at 6th and 24th hour (*P* < 0.01), and less than in the control group at 9th hour (*P* < 0.05). Besides, there was a significant difference in iron content of *1R* group and Δ*irp2* group (*P* < 0.01), on the 3rd, 6th, and 9th hour (Fig. 2). The results showed that *irp2* gene could mediate the iron absorption ability of *E. coli*, which was a great threat to cell growth.

## Discussion

The CRISPR/Cas9 gene editing system mainly depends on vector resistance and the specificity of *Cas9* nuclease expressed by sgRNA (Zerbini et al. 2017; Jiang et al. 2013). Because the vector carries resistance genes, positive clones can be screened from the resistance plate to decide whether the vector has been transferred to the target bacteria. It should be noted that in the gene editing of resistant strains, the selection of positive clones should fully consider the resistance genes carried by the selected vectors and the drug resistance of the strains. Therefore, from 41 wild-type *E. coli* screened 4 strains which were sensitive to Ampicillin and Kanamycin for gene editing. SgRNA is a special sequence with a length of about 20 bp and its 3′ end is close to the PAM sequence. It can complement and pair with the target site of genomic DNA as well as can also perform imperfect complementary pairing (off-target) with thousands of other sequences in the genome (Kleinstiver et al. 2015; Hsieh-Feng and Yang 2020). Therefore, when designing sgRNA of genomic target sites, we need to find PAM sequences in the target DNA that can be recognized by Cas9. In addition, the designed sgRNA can be used for wild-type *E. coli irp2* gene knockout, we need to obtain the *irp2* gene sequence of the knock-out strains, and find the wild strains and *E. coli* engineered strains *irp2* gene (the online addresses for sgRNA were designed using *E. coli* engineered bacterial genomes as templates) conserved region was used as template to design sgRNA. Finally, it is necessary to use software to check the specificity and potential off-target sites of gRNA. In general, we should consider our experimental materials and experimental conditions, select the proper expression vector (plasmid), and the specificity of sgRNA to make the efficiency of gene editing.

The editing of the target gene is of great significance to the study of its role. At present, genome editing technology based on CRISPR/Cas9 system is favored (Pyne et al. 2015; Sun et al. 2017), which has been applied to gene function research of zebrafish (Chang et al. 2013; Li et al. 2016), Arabidopsis (Zhang et al. 2018), *Saccharomyces cerevisiae* (DiCarlo et al. 2013; Mans et al. 2015) and other organisms. CRISPR/Cas9 system produces double strands breaks (DSBs) in target genes by *Cas9* nuclease, and then cells repair the damaged DNA through NHEJ or HDR pathway. NHEJ, as the main repair method of cells, can occur in any stage of the cell cycle. This repair method usually results in random insertion or deletion of several to hundreds of bases near the double-strand breaks, resulting in gene knockout (Hayashi and Tanaka 2019). In this research, three knock-out target sites were screened based on the *irp2* gene sequence, one in the sense chain and the other two in the anti-sense chain. Three expression vectors were constructed according to three different gene knock-out target sites, then the expression vectors and pCas vector were transformed to the target strains. According to the sequencing results, there were base mutations and deletion in the designed sense chain target sites, indicating that one of the 3 vectors played a role and the other two expression vectors were out of the target. The reasons for off-target were related to the specificity of sgRNA, the concentration of Cas9-sgRNA, and the mismatch between DNA and RNA. In the process of gene editing by CRISPR/Cas9 gene editing technology, there will be off-target phenomenon. Therefore, we can select several more sites and choose sgRNA with high specificity when selecting target sites, the concentration of Cas9-sgRNA was optimized and a suitable expression vector was selected. At the same time, it may be necessary to consider the compatibility of the introduced exogenous expression plasmid with the endogenous plasmid of the target strain and whether the CRISPR/Cas9 system originally contained in the strain will affect the effect of the exogenous expression plasmid. For example, since *Campylobacter jejuni* contains the CRISPR/Cas9 system, gene editing of this bacterium using CRISPR/Cas9 gene editing technology requires the consideration of suppressing the activity of the Cas9 nuclease it contains to achieve the desired gene editing result. On the contrary, the CRISPR/Cas system in *E. coli* is type I-E, and the CRISPR/Cas system of this type is inhibited by the DNA-binding protein H-NS (histone like nucleoid structure protein) when performing gene shearing function (Kiro et al. 2013). Therefore, when using CRISPR/Cas9 gene editing technology to edit *E. coli* genes, there is no need to consider the role of the CRISPR/Cas system. Only if all the above issues are fully considered and analyzed, then CRISPR/Cas9 gene editing technology will be more efficient when applied to gene editing of wild-type strains.

With the development of molecular biology technology, there have been many improvements in CRISPR/Cas9 technology. Jiang et al. (2015) introduced a homologous recombination system in Cas9 plasmid construction, while the donor sequence was later co-transferred into *E. coli MG*1655 with the expression plasmid to enable the CRISPR/Cas9 system to knock out several genes in this bacterium. The important improvement of this system is that the donor sequence does not need to be cloned inside the vector and is directly co-transformed with the plasmid, thus reducing the difficulty of the experiment. Wang et al. (2018) transferred pCas9 and pTargetF + donor plasmids into *E.coli BL*21, which not only achieved point mutation of the *proB* gene, but also avoid the risk of repeated cutting by gRNA/Cas9 without altering the PAM or inserting additional silent mutations into the genome. Li et al. (2021) modified the pCas/pTargetF system, CRISPR/Cas9 technology can be applied not only for gene editing of *E. coli BL*21(*DE*3), K-12 strains *MG*1655, *DH5α*, *CGMCC*3705 and other strains, but also to shorten the time to edit bacterial genomes. However, these studies were conducted on the genome of genetically engineered bacteria, not wild-type *E. coli*.

Existing online sgRNA design tools are not specifically targeted at wild-type *E. coli*. The evaluation of sgRNA off-target probability is based on *E. coli K*12, *MG*1655, *BL*21(*DE*3) instead of wild-type *E. coli*. It might lead to inaccurate evaluation of sgRNA off-target probability (Hou et al. 2020). So the sequences of wild-type *E. coli irp2* gene need to be compared with those of *E. coli* using the online sgRNA design tool, and 3 sgRNA sequences were designed according to their conserved region sequences. In addition, given the different resistance phenotypes of wild-type *E. coli*, strains need to be screened for antibiotic sensitivity for gene editing. Therefore, 4 strains that were sensitive to both ampicillin and kanamycin were selected for gene knockout according to the resistance of the CRISPR/Cas9 system expression vectors. Finally, the *irp2* gene was knocked out in three quarters of the wild-type *E. coli* strains with CRISPR/Cas9 technology combined with homologous recombination technology, which contained recombinant vectors with Cas9, sgRNA and donor sequences. From our current experimental results, the knock-out strategy can meet our requirements for gene editing of wild-type *E. coli*. In future research, it is still needed to expand the genome data of strains in the online sgRNA database, and modify the drug-resistant phenotype of wild-type strains according to the resistance of the expression vectors, so that the improved CRISPR/Cas9 system can be used for gene editing of more wild-type strains.

The *irp2* gene is a marker gene of HPI, which is closely related to pathogenicity and virulence. As mentioned in the introduction, deletion of the *irp2* gene results in the inability of the strain to synthesize Ybt and reduced virulence. Therefore, it has potential application values for the research of *irp2* gene function. To explore the function of *irp2* gene in wild-type *E. coli*, the Δ*irp2* strains were constructed by the CRISPR/Cas9 gene editing system, and the growth curves of the wild-type *1R* and Δ*irp2* strains were measured. The growth curve showed that the delay, logarithm and stable phase of the *1R* and Δ*irp2* strains were the same, indicating that *irp2* gene is not the key gene for the growth of *E. coli*. The results of iron absorption test and quantitative detection of iron carrier production showed that *irp2* gene could regulate the ability of *E. coli* to absorb iron and produce iron carrier, these results are of great significance for the research of the function of *irp2* gene in *E. coli*. This work was the first to knock out the *irp2* gene of *E. coli* from wild-type Saba pigs, which not only laid a foundation for the application of CRISPR/Cas9 gene editing technology in microbial gene editing but also provided a certain reference basis for the study of *irp2* gene function.

## Data Availability

All data generated or analyzed during this study are included in this published article.

## References

[CR1] Altenbuchner J (2016). Editing of the *Bacillus subtilis* genome by the CRISPR-Cas9 system. Appl Environ Microb.

[CR2] Benedek O, Schubert S (2007). Mobility of the *Yersinia* high-pathogenicity island (HPI): transfer mechanisms of pathogenicity islands (PAIS) revisited (a review). Acta Microbiol Immunol Hung.

[CR3] Chang N, Sun C, Gao L, Zhu D, Xu X, Zhu X, Xiong JW, Xi JJ (2013). Genome editing with RNA-guided Cas9 nuclease in zebrafish embryos. Cell Res.

[CR4] Cho JS, Choi KR, Prabowo CPS, Shin JH, Yang DS, Jang JD, Lee SY (2017). CRISPR/Cas9-coupled recombineering for metabolic engineering of *Corynebacterium glutamicum*. Metab Eng.

[CR5] Demirci Y, Zhang B, Unver T (2018). CRISPR/Cas9: an RNA-guided highly precise synthetic tool for plant genome editing. J Cell Physiol.

[CR6] DiCarlo JE, Norville JE, Mali P, Rios X, Aach J, Church GM (2013). Genome engineering in *Saccharomyces Cerevisiae* using CRISPR-Cas systems. Nucleic Acids Res.

[CR7] Feng Z, Zhang B, Ding W, Liu X, Yang DL, Wei P, Cao F, Zhu S, Zhang F, Mao Y, Zhu JK (2013). Efficient genome editing in plants using a CRISPR/Cas system. Cell Res.

[CR8] Hayashi A, Tanaka K (2019). Short-homology-mediated CRISPR/Cas9-based method for genome editing in fission yeast. G3 Genes Genome Genet.

[CR9] Hou M, Sun S, Feng Q, Dong X, Zhang P, Shi B, Liu J, Shi D (2020). Genetic editing of the virulence gene of *Escherichia coli* using the CRISPR system. PeerJ.

[CR10] Hsieh-Feng V, Yang Y (2020). Efficient expression of multiple guide RNAs for CRISPR/Cas genome editing. aBIOTECH.

[CR11] Jiang W, Bikard D, Cox D, Zhang F, Marraffini LA (2013). RNA-guided editing of bacterial genomes using CRISPR-Cas systems. Nat Biotechnol.

[CR12] Jiang Y, Chen B, Duan C, Sun B, Yang J, Yang S (2015). Multigene editing in the *Escherichia coli* genome via the CRISPR-Cas9 system. Appl Environ Microbiol.

[CR13] Kiro R, Goren MG, Yosef I, Qimron U (2013). CRISPR adaptation in *Escherichia coli* subtypeI-E system. Biochem Soc Trans.

[CR14] Kleinstiver BP, Prew MS, Tsai SQ, Topkar VV, Nguyen NT, Zheng Z, Gonzales AP, Li Z, Peterson RT, Yeh JR, Aryee MJ, Joung KJ (2015). Engineered CRISPR-Cas9 nucleases with altered PAM specificities. Nature.

[CR16] Li M, Zhao L, Page-McCaw PS, Chen W (2016). Zebrafish genome engineering using the CRISPR-Cas9 system. Trends Genet.

[CR17] Li Q, Sun B, Chen J, Zhang Y, Jiang Y, Yang S (2021). A modified pCas/pTargetF system for CRISPR-Cas9-assisted genome editing in *Escherichia coli*. Acta Biochim Biophys Sin (shanghai).

[CR101] Lian LS, Lu SX, Yan DW (2006) The breeding of Saba pig synthetic line[C]. World Congress on Genetics Applied to Livestock Production

[CR18] Magistro G, Magistro C, Stief CG, Schubert S (2017). The high-pathogenicity island (HPI) promotes flagellum-mediated motility in extraintestinal pathogenic *Escherichia coli*. PLoS One.

[CR19] Mans R, van Rossum HM, Wijsman M, Backx A, Kuijpers NG, van den Broek M, Daran-Lapujade P, Pronk JT, van Maris AJ, Daran JM (2015). CRISPR/Cas9: a molecular Swiss army knife for simultaneous introduction of multiple genetic modifications in *Saccharomyces cerevisiae*. FEMS Yeast Res.

[CR20] Messerer M, Fischer W, Schubert S (2017). Investigation of horizontal gene transfer of pathogenicity islands in *Escherichia coli* using next-generation sequencing. PLoS One.

[CR21] Pyne ME, Moo-Young M, Chung DA, Chou CP (2015). Coupling the CRISPR/Cas9 system with lambda red recombineering enables simplified chromosomal gene replacement in *Escherichia coli*. Appl Environ Microbiol.

[CR100] Rakin A, Garzetti D (2013). Different siderophores contribute to the high-pathogenicity phenotype in Yersinia. Probl Particularly Dangerous Infect.

[CR22] Schwyn B, Neilands JB (1987). Universal chemical assay for the detection and determination of siderophores. Anal Biochem.

[CR23] Smati M, Magistro G, Adiba S, Wieser A, Picard B, Schubert S, Denamur E (2017). Strain-specific impact of the high-pathogenicity island on virulence in extra-intestinal pathogenic *Escherichia coli*. Int J Med Microbiol.

[CR24] Sun L, He T, Zhang L, Pang M, Zhang Q, Zhou Y, Bao H, Wang R (2017). Generation of newly discovered resistance gene *mcr-1* knockout in *Escherichia coli* using the CRISPR/Cas9 system. J Microbiol Biotechnol.

[CR25] Tu J, Xue T, Qi K, Shao Y, Huang B, Wang X, Zhou X (2016). The *irp2* and *fyuA* genes in High Pathogenicity Islands are involved in the pathogenesis of infections caused by avian pathogenic *Escherichia coli* (APEC). Pol J Vet Sci.

[CR26] Tu J, Qi K, Song X, Xue T, Ji H, Shao Y, Liu H, Zhou X, Zhu L (2017). Horizontal transfer and functional evaluation of high pathogenicity islands in Avian *Escherichia coli*. Pol J Vet Sci.

[CR27] Urnov FD, Rebar EJ, Holmes MC, Zhang HS, Gregory PD (2010). Genome editing with engineered zinc finger nucleases. Nat Rev Genet.

[CR28] Wang X, He J, Le K (2018). Making point mutations in *Escherichia coli BL21* genome using the CRISPR-Cas9 system. FEMS Microbiol Lett.

[CR29] Wiedenheft B (2013). In defense of phage: viral suppressors of CRISPR-mediated adaptive immunity in bacteria. RNA Biol.

[CR30] Yang J, Meng X, Pan J, Jiang N, Zhou C, Wu Z, Gong Z (2018). CRISPR/Cas9-mediated noncoding RNA editing in human cancers. RNA Biol.

[CR31] Zerbini F, Zanella I, Fraccascia D, König E, Irene C, Frattini LF, Tomasi M, Fantappiè L, Ganfini L, Caproni E, Parri M, Grandi A, Grandi G (2017). Large scale validation of an efficient CRISPR/Cas-based multi gene editing protocol in *Escherichia coli*. Microb Cell Fact.

[CR32] Zhang Y, Ma X, Xie X, Liu YG (2017). CRISPR/Cas9-based genome editing in plants. Prog Mol Biol Transl Sci.

[CR33] Zhang Q, Xing HL, Wang ZP, Zhang HY, Yang F, Wang XC, Chen QJ (2018). Potential high-frequency off-target mutagenesis induced by CRISPR/Cas9 in Arabidopsis and its prevention. Plant Mol Biol.

